# Trends and forecasted rates of adverse childhood experiences among adults in the United States: an analysis of the Behavioral Risk Factor Surveillance System

**DOI:** 10.1515/jom-2022-0221

**Published:** 2023-03-22

**Authors:** Micah Hartwell, Amy Hendrix-Dicken, Rachel Terry, Sadie Schiffmacher, Lauren Conway, Julie M. Croff

**Affiliations:** Oklahoma State University College of Osteopathic Medicine at Cherokee Nation, Office of Medical Student Research, Tahlequah, OK, USA; Department of Paediatrics, University of Oklahoma, School of Community Medicine, Tulsa, OK, USA; Oklahoma State University College of Osteopathic Medicine, Office of Medical Student Research, Tulsa, OK, USA; Oklahoma State University College of Osteopathic Medicine at Cherokee Nation, Office of Medical Student Research, Tahlequah, OK, USA; Department of Paediatrics, University of Oklahoma, School of Community Medicine, Tulsa, OK, USA; Oklahoma State University Centre for Health Sciences, National Centre for Wellness and Recovery, Tulsa, OK, USA

**Keywords:** adverse childhood events, adversity, BRFSS, trauma

## Abstract

**Context:**

Many studies have shown increases in negative social aspects in the United States that may increase the likelihood of a child experiencing adversity. These rising trends include household dysfunction, poor mental health and substance use, crime rates, and incarceration. Additionally, the pathway of adverse childhood experiences (ACEs) may also perpetuate intergenerational trauma.

**Objectives:**

Given these increased trends, our objective was to determine the mean ACEs reported among adults by year of birth to assess trends of ACEs over time.

**Methods:**

To assess ACEs trends in the United States, we utilized data from the 2020 Behavioral Risk Factor Surveillance System (BRFSS), a nationally representative survey. We summed individuals’ reported ACEs and then calculated the mean ACE score within age cohorts (in 1-year increments) by year of birth. We utilized an auto-regressive integrated moving average (ARIMA) model to forecast mean ACEs through 2030.

**Results:**

Respondents to the ACEs module (n=116,378) represented 63,076,717 adults in the United States, with an average age cohort of 1715 individuals. The mean reported ACEs among individuals 80 years or older (born in or before 1940) was 0.79, while the highest mean ACEs (2.74) were reported among the cohort born in 1998—an average increase of 0.022 ACEs per year. The ARIMA model forecasted that individuals born in 2018 will, on average, surpass a cumulative of three ACEs.

**Conclusions:**

Given the connection of ACEs to poor health outcomes and quality of life, this trend is alarming and provides evidence for the necessity of child maltreatment prevention. Multigenerational trauma-informed care and education are warranted for individuals with ACEs and may even prevent the cycle from recurring.

The Adverse Childhood Experiences (ACEs) study was one of the first to demonstrate the robust, life-long effects of maltreatment, including physical, sexual, and emotional abuse, and household dysfunction, including domestic violence, parental divorce, and living with an adult with substance misuse or who is or has been incarcerated during childhood [1]. Although previous research has shown that ACE scores may not always be predictive of adverse health outcomes at the individual level, ACEs screeners remain a powerful tool for researchers and clinicians to advocate for awareness and mitigation of the population-level harms of childhood adversity [2].

Many studies have shown increases in negative social aspects in the United States that may increase the likelihood of a child experiencing these categories of adversity. First, previous reports have shown increasing trends within the domains of household dysfunction—especially among incarceration and substance misuse. As noted in *The Growth of Incarceration in the United States: Exploring Causes and Consequences*, the nation’s incarceration rate has more than quadrupled in the past 40 years [3]. These incarceration rates stem from large increases in violent, property, and drug crimes from 1960, with the two former types peaking in the 1990s, whereas the latter remained increasing through the 2010s [3]. Growth in the trends of drug arrests coincides with the new types of drugs introduced to the supply chain including marijuana, heroin, cocaine, methamphetamine, and synthetic opioids, and their usage [4]. Analysis from the National Household Survey on Drug Abuse shows that prior to World War II, only alcohol and cigarettes were commonly utilized among the US population; however, following the war, the use of other substances, including those previously mentioned, became much more prevalent [5].

Additionally, from 1960 to 1990, there was a substantial decrease in the number of two-parent (biological parent) households, declining approximately 20%—from 70.6 to 51.0% [6, 7]. A 2019 report estimates that the number of children living with only one parent is approximately 23.2% [8], with nearly 47.1% of single mothers living below the poverty threshold and 23.0% of single fathers below the threshold [9]. Lower-income households are more often subjected to inequities and poorer physical and social environments, including food insecurity, neighborhood crime, and lack of access to resources and support [10]. Further, parents who have experienced multiple ACEs may also exhibit a lack of sensitivity toward their children, an increased risk of perpetrating abuse, and have disruptive parent-child interactions [11]. ACEs among parents are also associated with poorer child health outcomes and academic performance, which, in turn, are also linked to ACEs [12]. Thus, this cyclical ACE pathway, rooted in attachment theory and developmental psychopathology [13], may perpetuate an increased risk for more ACEs in future generations [14].

Given the prevalence of ACEs reported by Felitti et al. [1] in the original ACEs study (1998) and several studies since [15, 16], in addition to the increasing risk factors for ACEs in the United States, we hypothesized that the mean ACE score among adults in the United States would show a rising trend over time. Thus, the objective of this study was to investigate trends in cumulative ACEs from 1940 through 2020 utilizing the Behavioral Risk Factor Surveillance System (BRFSS). Further, we sought to determine if and when US adults would surpass a mean of three ACEs, which is considered the threshold for a minimal risk of adverse health outcomes [17], given previous research indicating four or more ACEs as the high-risk threshold for population-level disparities in comorbid diseases and health outcomes including, but not limited to, pulmonary disease, cardiovascular disease, cancer, depression, substance use, and suicide [18], and disrupted education [12].

## Methods

### Design, data, and participation

To assess trends in ACEs among adults in the United States, we performed a cross-sectional analysis utilizing data from the 2020 cycle of the BRFSS. The BRFSS is a nationally representative survey performed in the United States. The ACE Module in the BRFSS was administered among 28 states and Washington, D.C. The module asks 11 questions pertaining to the childhood experiences of domestic violence, parental divorce, living with adults who experienced incarceration, mental health disorders or substance use, or emotional, physical, or sexual abuse (Supplementary 1). The BRFSS ACE module was adapted from the original ACE study, which included 17 items. The reduction of items, done in collaboration with authors from the original study and other experts, was to make the collection of ACEs appropriate for the largescale, phone-based survey. The constructs of the original survey remained intact [19], and the BRFSS ACE module has shown similar results in terms of: (1) cumulative scores; and (2) the relationship between ACEs and health—with four or more cumulative ACEs being associated with multiple adverse outcomes [20].

### Measures and statistical analysis

First, we report the number of individuals (n) responding to the ACEs module and population estimate (N) by applying the survey design and sampling weights provided by BRFSS. The complex survey design through BRFSS accounts for demographics, including age, education, race/ethnicity, and so on, through oversampling and the use of data weighting [21]—which were employed within our study. To assess the historical trends of cumulative ACEs within the US population, we first summed the number of ACE items each participant reported to have occurred to calculate their ACE score. To create our age cohorts (in 1-year increments), we determine participants’ year of birth by subtracting the reported age in BRFSS from the survey year (2020). Then, we calculated the mean ACE score reported by individuals within the participant’s age cohort. Utilizing the history (age cohorts) of mean ACEs, we calculated the linear trend of the mean ACEs and then utilized an auto-regressive integrated moving average (ARIMA) to forecast mean ACEs through 2030 to determine the year of birth when US residents may surpass a mean of three cumulative ACEs. Statistical analyses were performed in R (v.4.0.4, R Core Team) utilizing the forecast package [22] to calculate the best-fitted ARIMA model. This project was submitted for ethics review and determined to be nonhuman subject research as defined in 45 CFR 46.102(d) and (f).

## Results

**Figure 1:**
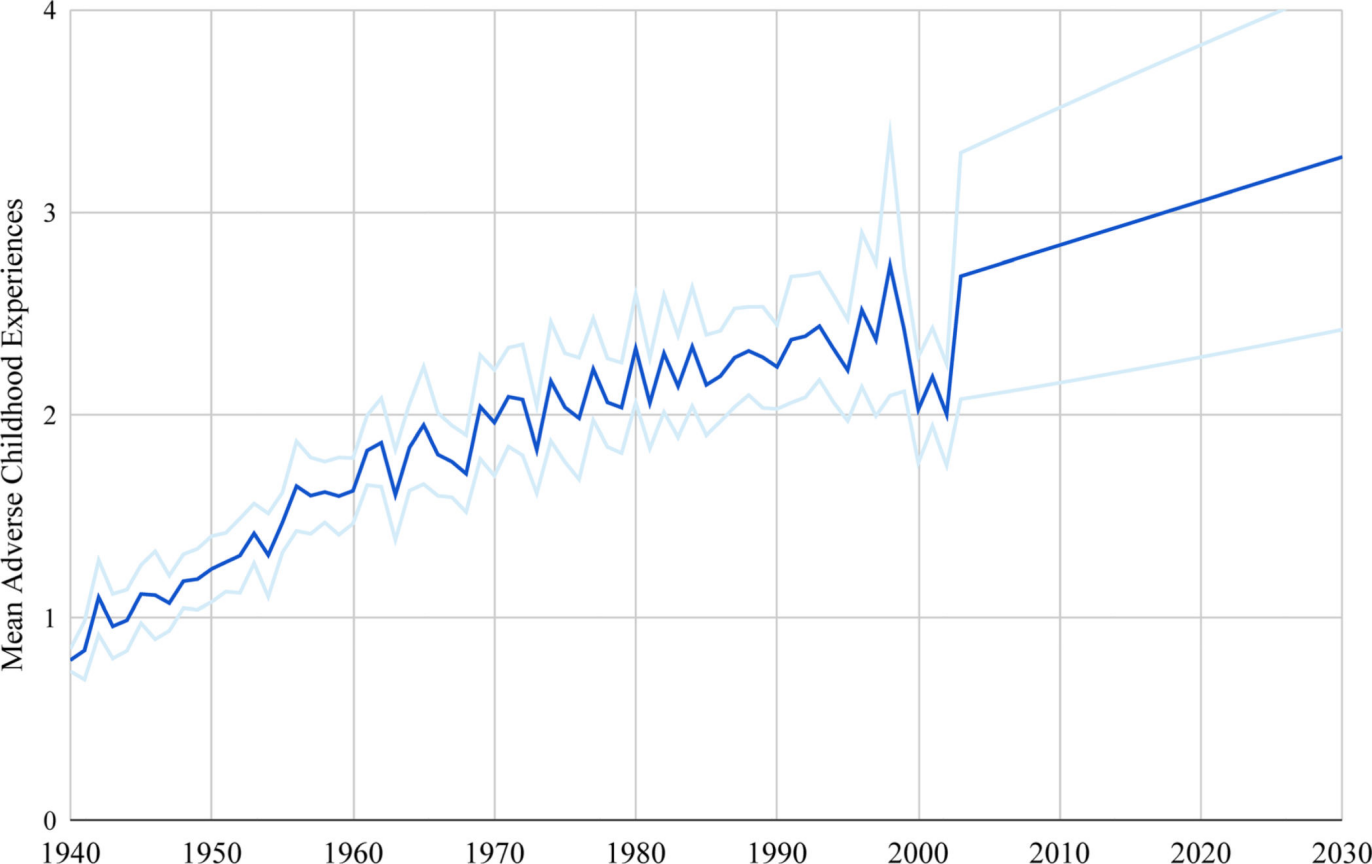
Mean adverse childhood experiences (ACEs) of respondents from the 2020 Behavioral Risk Factor Surveillance System (BRFSS) aged 18–80+ plotted by year of birth in the United States with forecasting to 2030.

From the 2020 BRFSS, 116,378 individuals provided responses to the ACEs module, representing 63,076,717 adults in the United States. The average sample size per 1-year age cohort (from 18 to 79) was 1714.6 (SD=535.9), and 10,071 respondents in the 80+ cohort (Supplement 2). The mean number of ACEs reported by participants 80 years or older (born in or before 1940) was 0.79 (95% CI: 0.74–0.85), whereas the highest number of ACEs (M=2.74; 95% CI: 2.09–3.38) were reported by respondents who were 22 years of age (born in 1998; Figure 1). Between 1940 and 2002, the mean ACE score among individuals in their respective cohort increased by 0.024 (SE=0.001) per year on average.

Utilizing the history of mean ACEs from 1940 through 2002, the best-fitted ARIMA model forecasted a linear trend from 2003 through 2030—increasing by 0.022 ACEs per year. The model shows that individuals born in 2018 will, on average, surpass a cumulative of three ACEs. A decrease in reported ACEs can be seen in the figure among individuals aged 18 to 20 (born from 2000 to 2002). Mean ACEs per cohort are provided in Supplement 2. ARIMA parameters p, d, and q were 0, 1, and 1, respectively, and allowed for drift. The model fit AICc was −58.73, and the Ljung–Box Q was =6.97 (*df*=10, p=0.54).

## Discussion

Our study found that, on average, the number of ACEs reported by adults in the US increased by 0.024 per year from 0.79 in 1940, with a peak among those born in 1998 at 2.74. Further, the predictive model forecasts that US children born in 2018 will, on average, experience more than three ACEs unless widespread intervention occurs. To our knowledge, this is the first study to assess the longitudinal increase of ACEs in this manner—utilizing the average of the adult participants’ ACE scores within their respective birth-year cohort. This is feasible due to the temporal nature of ACEs—events that occurred before the age of 18. Further, this assessment was conducted with a nationally representative dataset and provides forecasted estimates of ACEs among future generations. The increasing trend of average ACE scores across the past 80 years supports behavioral and biological theories regarding the transmission of intergenerational trauma. Our findings are contrary to a previous publication, which suggests that ACEs are declining via the assessment of risk factors [23]; however, this study by Finkelhor [23] includes multiple ACEs that were not in the same domains as the original ACEs study nor within the BRFSS module, such as the severe illness of the individual, or illness or death of a parent or sibling—in addition to a different methodological approach. The decrease in reported ACEs among 18- to 20-year-olds is of interest. The lower reported ACEs among these individuals may be due to under-reporting because they may likely still live at home. Although more speculatively, it may also be due to increased prevention of child abuse and neglect, as the early 1990s saw the expansion of family support services with the authorization of the Family Preservation and Support Services Program Act, which increased funding for support services by close to $1 billion over five years [24].

Implementation of screening and resilience practices as a two-generational approach is necessary in order to prevent the continued intergenerational accumulation of ACEs and associated mental and physical comorbidities [25]. CDC Guidelines for Preventing ACEs include strengthening economic support to families, teaching skills (social-emotional learning, parenting, healthy relationships) to families, and early intervention by trauma-informed primary care and services for those impacted by trauma to affect this trajectory [26]. Addressing ACEs in this fashion can help connect parents to appropriate resources for their own trauma, and help them develop healthy appropriate parenting practices that they may not have experienced as a child. This is crucial because an expanding body of knowledge has shown the impact that positive parent-child relationships can have regarding the prevention and protection against early childhood adversity [27–31]. Because not all children exposed to ACEs experience poor outcomes, future research should include measures and interventions of protective factors associated with resilience to determine if they too are increasing over time, thus mitigating the effect of adversity in children experiencing increased ACEs [32]. Unfortunately, there are a variety of reasons why two-generational approaches are not widely available or may fail. As Shonkoff and Fisher [33] explain, many of these programs are time-intensive and costly, making them diffcult to implement on a wide scale. The level of funding needed as well as the conflicting analysis of programs can make policy makers hesitant to fund such initiatives [33].

Given the projected increase in average ACE scores for individuals living in the United States, it is imperative that appropriate interventions and response frameworks be established now. Population-level health analyses have shown a link between ACEs and costly chronic health conditions as well as risky behaviors that can cause deleterious health outcomes [18, 34]. These chronic conditions, such as cardiovascular disease, obesity, and diabetes, among others, create an increased healthcare burden for both patients and healthcare systems [18, 34]. Whereas the ACEs questionnaire has allowed the conversation surrounding trauma’s impact on health to become more accessible to the general public, widespread prevention efforts in the US healthcare system are still lacking. One problem commonly encountered is the ability to not only screen children and their families appropriately but also to respond to information that is disclosed during the screening in the limited clinical visit time that most providers encounter. Collaborative multidisciplinary teams appear to provide the resources, including time, for providers to utilize ACE questionnaires as part of routine clinical assessment. For instance, Liu et al. [35] found that approaching ACEs with a collaborative practice model (CPM) was highly successful in the ability to screen individuals of a diverse patient population as well as respond to disclosures made by families. This model included a clinical care manager, licensed social worker, family support specialist (a parental peer), and a child psychiatrist available for consultations as part of the child’s care team [35].

While the CPM provides a framework that would be ideal, in reality, many physicians do not have access to team-based care. It is not feasible for physicians alone to identify and manage all aspects of childhood adversity; however, they can incorporate trauma-informed care into their practices. The Substance Abuse and Mental Health Services Agency (SAMHSA) has outlined six key principles for this type of care delivery, including “(1) safety, (2) trustworthiness and transparency, (3) peer support, (4) collaboration and mutuality, (5) empowerment, voice, and choice, and (6) cultural, historical, and gender issues [36]”. This approach to care is consistent with the four tenets of osteopathic medicine as pointed out by Marcoux [37]. By shifting focus to all potential drivers of health, including ACEs, physicians will be better equipped to meet the unique healthcare needs of their patients [37]. Tools like ACEs Aware’s extensive guide titled “ACEs Aware Trauma-Informed Network of Care Roadmap” are great resources that can help physicians determine what they can feasibly incorporate into their own practices while also identifying services available to their patients. This guide outlines specific clinical and nonclinical resources and infrastructure needed for the purpose of trauma-informed care, which includes identifying the risk for toxic stress in children as well as clinical response and follow-up procedures [38].

### Implementing practice changes

Regardless of the approach a physician may elect to take, there are several important considerations prior to implementing a trauma-informed care approach or ACEs screening. First, physician education is paramount to successfully implementing any of the above. Physicians should utilize services and education materials provided by groups like SAMHSA and Indian Health Services (IHS), which mandates trauma-informed care training for all of its employees [39, 40]. IHS offers these on-demand publicly accessible trainings for no cost (https://www.ihs.gov/teleeducation/training/). Physicians must also be trained to prevent re-traumatization and appropriate follow-up of ACE disclosures [41]. The implementation of full-scale screening for ACEs in primary care is still relatively new. A recent publication by Ridout et al. [41] (which includes Dr. Felitti, who conducted the first ACEs study) approaches screening from a clinical processes prospective, and offers valuable insight for anyone interested in implementing an ACEs screen into their practice model. Ridout et al. [41] proposes a simplified version of the ACEs screen for the sake of clinical process disruption; however, providers should be mindful that screens that have not been validated via extensive study may lack the validity and specificity of validated versions [41]. Physicians should keep in mind that many ACEs screens do not include specific questions related to racism, historical trauma, or trauma related to being a refugee of war [42, 43]. As such, sole reliance on the screen for consideration of all trauma is inappropriate. ACEs Aware provides a list of potential screeners to utilize, including the Pediatric ACEs and Related Life Events Screener (PEARLS), which does cover some of these topics [44].

### Limitations

Limitations of our study include the potential of generational effects, memory, or response bias, because the ACEs were retrospectively self-reported; however, self-reported ACEs have shown general validity [45] and likely did not affect the trend because the trajectory continues through younger cohorts (20–65 years of age). Further, there is not one universally accepted ACE assessment tool; thus, other tools may vary in the responses. Additionally, selection bias and social desirability bias are other potential limitations within self-report surveys. However, the large sample size—a strength of this study—would likely counter these biases.

## Conclusions

Our findings show an increasing trend of ACEs in the United States among children born from 1940 to 2002. Further, the projected birth year in which children would surpass three ACEs was 2018. Given the linkage of ACEs to poor health outcomes and quality of life, the trend is alarming and provides evidence that increased child maltreatment prevention is necessary. Incorporating multi-generational trauma-informed care and education for individuals with ACEs is warranted and may prevent the cycle from recurring.

## Supplementary Material

Supplementary Material

**Supplementary Material:** This article contains supplementary material (10.1515/jom-2022-0221).
