# Supplementary material for: Trends and forecasted rates of adverse childhood experiences among adults in the United States: an analysis of the Behavioral Risk Factor Surveillance System
Source: J Osteopath Med. Author manuscript; Available in PMC 2025 Jun 21. (PMC12181775; doi:10.1515/jom-2022-0221)
Supplement: Supplementary Material [file NIHMS2074379-supplement-Supplementary_Material.docx]

Supplement 1. Questions within BRFSS ACEs Module.

| Question 1. | Did you live with anyone who was depressed, mentally ill, or suicidal? |
| --- | --- |
| Question 2. | Did you live with anyone who was a problem drinker or alcoholic? |
| Question 3. | Did you live with anyone who used illegal street drugs or who abused prescription medications? |
| Question 4. | Did you live with anyone who served time or was sentenced to serve time in a prison, jail, or other correctional facility? |
| Question 5. | Were your parents separated or divorced? |
| Question 6. | How often did your parents or adults in your home ever slap, hit, kick, punch or beat each other up? |
| Question 7. | Not including spanking, (before age 18), how often did a parent or adult in your home ever hit, beat, kick, or physically hurt you in any way? |
| Question 8. | How often did a parent or adult in your home ever swear at you, insult you, or put you down? |
| Question 9. | How often did anyone at least 5 years older than you or an adult, ever touch you sexually? |
| Question 10. | How often did anyone at least 5 years older than you or an adult, try to make you touch them sexually? |
| Question 11. | How often did anyone at least 5 years older than you or an adult, force you to have sex? |

| Supplement 2. Mean adverse childhood experiences (ACEs) reported in BRFSS by age of participants. | | | | | | | | | |
| --- | --- | --- | --- | --- | --- | --- | --- | --- | --- |
| Age | Calculated Year of birth | Sample size (n) | Population Estimate (N) | ACEs | | ARIMA Forecasted Model  to Birth Year of 2030 | | | |
|  |  |  |  | Mean | 95% CI | Age | Year of birth | ACEs | |
| 80+ | 1940 | 10,071 | 3,214,696 | 0.79 | 0.74 - 0.85 |  |  | Mean | 95% CI |
| 79 | 1941 | 1,425 | 491,916 | 0.84 | 0.69 - 0.98 | 17 | 2003 | 2.69 | 2.08 - 3.29 |
| 78 | 1942 | 1,589 | 515,907 | 1.10 | 0.92 - 1.28 | 16 | 2004 | 2.71 | 2.09 - 3.33 |
| 77 | 1943 | 1,792 | 656,254 | 0.96 | 0.8 - 1.12 | 15 | 2005 | 2.73 | 2.10 - 3.36 |
| 76 | 1944 | 1,785 | 592,809 | 0.99 | 0.84 - 1.14 | 14 | 2006 | 2.75 | 2.11 - 3.39 |
| 75 | 1945 | 1,946 | 660,055 | 1.12 | 0.97 - 1.26 | 13 | 2007 | 2.77 | 2.12 - 3.42 |
| 74 | 1946 | 1,952 | 692,934 | 1.11 | 0.89 - 1.33 | 12 | 2008 | 2.79 | 2.13 - 3.45 |
| 73 | 1947 | 2,493 | 928,055 | 1.07 | 0.94 - 1.21 | 11 | 2009 | 2.82 | 2.15 - 3.49 |
| 72 | 1948 | 2,527 | 882,558 | 1.18 | 1.05 - 1.31 | 10 | 2010 | 2.84 | 2.16 - 3.52 |
| 71 | 1949 | 2,315 | 825,716 | 1.19 | 1.04 - 1.34 | 9 | 2011 | 2.86 | 2.17 - 3.55 |
| 70 | 1950 | 2,724 | 951,847 | 1.24 | 1.08 - 1.40 | 8 | 2012 | 2.88 | 2.18 - 3.58 |
| 69 | 1951 | 2,493 | 838,711 | 1.27 | 1.13 - 1.42 | 7 | 2013 | 2.9 | 2.20 - 3.61 |
| 68 | 1952 | 2,625 | 903,079 | 1.31 | 1.12 - 1.49 | 6 | 2014 | 2.93 | 2.21 - 3.64 |
| 67 | 1953 | 2,592 | 862,381 | 1.42 | 1.27 - 1.56 | 5 | 2015 | 2.95 | 2.22 - 3.67 |
| 66 | 1954 | 2,496 | 961,828 | 1.31 | 1.10 - 1.51 | 4 | 2016 | 2.97 | 2.23 - 3.70 |
| 65 | 1955 | 2,838 | 1,033,575 | 1.47 | 1.32 - 1.62 | 3 | 2017 | 2.99 | 2.25 - 3.73 |
| 64 | 1956 | 2,420 | 1,125,140 | 1.65 | 1.43 - 1.87 | 2 | 2018 | 3.01 | 2.26 - 3.76 |
| 63 | 1957 | 2,438 | 1,282,926 | 1.60 | 1.41 - 1.79 | 1 | 2019 | 3.03 | 2.27 - 3.80 |
| 62 | 1958 | 2,522 | 1,200,962 | 1.62 | 1.47 - 1.77 | - | 2020 | 3.06 | 2.29 - 3.83 |
| 61 | 1959 | 2,040 | 1,021,370 | 1.60 | 1.41 - 1.79 | - | 2021 | 3.08 | 2.30 - 3.86 |
| 60 | 1960 | 2,549 | 1,228,576 | 1.63 | 1.46 - 1.79 | - | 2022 | 3.1 | 2.31 - 3.89 |
| 59 | 1961 | 2,077 | 1,064,259 | 1.83 | 1.65 - 2.00 | - | 2023 | 3.12 | 2.33 - 3.92 |
| 58 | 1962 | 2,160 | 1,071,235 | 1.86 | 1.65 - 2.08 | - | 2024 | 3.14 | 2.34 - 3.95 |
| 57 | 1963 | 2,011 | 973,169 | 1.61 | 1.38 - 1.83 | - | 2025 | 3.16 | 2.35 - 3.98 |
| 56 | 1964 | 1,999 | 968,527 | 1.84 | 1.63 - 2.06 | - | 2026 | 3.19 | 2.37 - 4.01 |
| 55 | 1965 | 2,035 | 1,094,634 | 1.95 | 1.66 - 2.24 | - | 2027 | 3.21 | 2.38 - 4.04 |
| 54 | 1966 | 1,832 | 1,021,215 | 1.81 | 1.60 - 2.01 | - | 2028 | 3.23 | 2.39 - 4.07 |
| 53 | 1967 | 1,756 | 1,016,116 | 1.77 | 1.59 - 1.95 | - | 2029 | 3.25 | 2.41 - 4.09 |
| 52 | 1968 | 1,766 | 1,000,688 | 1.71 | 1.52 - 1.90 | - | 2030 | 3.27 | 2.42 - 4.12 |
| 51 | 1969 | 1,727 | 1,004,359 | 2.04 | 1.78 - 2.30 |  |  |  |  |
| 50 | 1970 | 2,025 | 1,222,135 | 1.96 | 1.70 - 2.22 |  |  |  |  |
| 49 | 1971 | 1,697 | 1,041,537 | 2.09 | 1.85 - 2.33 |  |  |  |  |
| 48 | 1972 | 1,616 | 973,903 | 2.08 | 1.80 - 2.35 |  |  |  |  |
| 47 | 1973 | 1,486 | 892,980 | 1.83 | 1.61 - 2.04 |  |  |  |  |
| 46 | 1974 | 1,447 | 833,780 | 2.17 | 1.87 - 2.46 |  |  |  |  |
| 45 | 1975 | 1,532 | 838,092 | 2.04 | 1.77 - 2.30 |  |  |  |  |
| 44 | 1976 | 1,345 | 1,027,009 | 1.98 | 1.68 - 2.28 |  |  |  |  |
| 43 | 1977 | 1,500 | 1,054,218 | 2.23 | 1.97 - 2.48 |  |  |  |  |
| 42 | 1978 | 1,596 | 1,042,208 | 2.06 | 1.84 - 2.28 |  |  |  |  |
| 41 | 1979 | 1,337 | 870,848 | 2.04 | 1.81 - 2.26 |  |  |  |  |
| 40 | 1980 | 1,705 | 1,246,069 | 2.33 | 2.06 - 2.60 |  |  |  |  |
| 39 | 1981 | 1,470 | 936,919 | 2.06 | 1.84 - 2.28 |  |  |  |  |
| 38 | 1982 | 1,549 | 1,091,446 | 2.30 | 2.01 - 2.59 |  |  |  |  |
| 37 | 1983 | 1,402 | 762,899 | 2.14 | 1.89 - 2.39 |  |  |  |  |
| 36 | 1984 | 1,432 | 1,016,530 | 2.34 | 2.04 - 2.63 |  |  |  |  |
| 35 | 1985 | 1,400 | 915,866 | 2.15 | 1.9 - 2.40 |  |  |  |  |
| 34 | 1986 | 1,317 | 1,216,714 | 2.19 | 1.97 - 2.42 |  |  |  |  |
| 33 | 1987 | 1,321 | 1,062,668 | 2.28 | 2.04 - 2.53 |  |  |  |  |
| 32 | 1988 | 1,306 | 983,160 | 2.32 | 2.10 - 2.53 |  |  |  |  |
| 31 | 1989 | 1,097 | 976,994 | 2.28 | 2.03 - 2.54 |  |  |  |  |
| 30 | 1990 | 1,327 | 1,110,285 | 2.24 | 2.03 - 2.44 |  |  |  |  |
| 29 | 1991 | 1,097 | 963,968 | 2.37 | 2.06 - 2.68 |  |  |  |  |
| 28 | 1992 | 1,169 | 926,657 | 2.39 | 2.09 - 2.69 |  |  |  |  |
| 27 | 1993 | 1,077 | 862,849 | 2.44 | 2.17 - 2.71 |  |  |  |  |
| 26 | 1994 | 985 | 797,366 | 2.33 | 2.06 - 2.59 |  |  |  |  |
| 25 | 1995 | 1,045 | 772,795 | 2.22 | 1.97 - 2.47 |  |  |  |  |
| 24 | 1996 | 1,026 | 1,099,357 | 2.52 | 2.14 - 2.90 |  |  |  |  |
| 23 | 1997 | 1,013 | 973,731 | 2.37 | 1.99 - 2.75 |  |  |  |  |
| 22 | 1998 | 991 | 1,023,824 | 2.74 | 2.09 - 3.38 |  |  |  |  |
| 21 | 1999 | 1,077 | 1,160,685 | 2.42 | 2.12 - 2.72 |  |  |  |  |
| 20 | 2000 | 975 | 1,072,161 | 2.03 | 1.77 - 2.29 |  |  |  |  |
| 19 | 2001 | 990 | 1,062,207 | 2.19 | 1.95 - 2.43 |  |  |  |  |
| 18 | 2002 | 1,031 | 1,159,357 | 2.00 | 1.75 - 2.25 |  |  |  |  |
